# Mitochondrial genomes of the South American electric knifefishes *Eigenmannia humboldtii* (Steindachner 1878), *Eigenmannia limbata* (Schreiner and Miranda Ribeiro 1903), *Sternopygus aequilabiatus* (Humboldt 1805) and *Sternopygus macrurus* (Bloch and Schneider 1801), (Gymnotiformes, Sternopygidae)

**DOI:** 10.1080/23802359.2018.1469386

**Published:** 2018-05-11

**Authors:** Melissa Rincón-Sandoval, Ricardo Betancur-R, Javier A. Maldonado-Ocampo

**Affiliations:** a Laboratorio de Ictiología, Unidad de Ecología y Sistemática -Unesis, Departamento de Biología, Facultad de Ciencias Pontificia, Universidad Javeriana, Bogotá, Colombia;; b Department of Biology, University of Puerto Rico – Río Piedras, San Juan, Puerto Rico

**Keywords:** Electric knifefishes, mitochondrial genome, genome-wide sequencing

## Abstract

We report four mitochondrial genomes of South American electric knifefishes, derived from target capture and Illumina sequencing (HiSeq 2500 PE100). Two trans-Andean species *Eigenmannia humboldtii* (mitochondrial consensus genome of 25 individuals) and *Sternopygus aequilabiatus* (mitochondrial consensus genome of 30 individuals) from Colombia and two cis-Andean species *Eigenmannia limbata* from Suriname and *Sternopygus macrurus* from Argentina. Regarding *Eigenmannia humboldtii*, *Eigenmannia limbata,* and *Sternopygus macrurus* mitochondrial genomes have 13 protein-coding genes, 1 D-loop, 2 ribosomal RNAs, 22 transfer RNAs, and are 13,394 bp, 10,921 bp, and 13,013 bp in length respectively, for *Sternopygus aequilabiatus* mitochondrial genomes have 13 protein-coding genes, 2 ribosomal RNAs, 22 transfer RNAs, and is 14,270 bp in length.

Family Sternopygidae conform a group of species commonly known as glass knifefishes that are broadly distributed throughout, most of the, Neotropics on both sides of the Andes, from Panama to northern Argentina (Albert 2001, 2003). This family comprises 6 genera (*Sternopygus, Eigenmannia, Rhabdolichops, Archolaemus, Distocyclus,* and *Japigny*) and 41 species (Eschmeyer et al. 2018). This study reports the complete mitochondrial genome for four different species belonging to the order Gymnotiformes, family Sternopygidae that has not been sequenced so far and provides important information for future studies.

In this work, 25 individuals, in total, of *Eigenmannia humboldtii* were collected in field at Atrato, Cauca, Magdalena, San Jorge, and Tuira rivers basins and 30 individuals, in total, of *Sternopygus aequilabiatus* were collected in field at Atrato, Cauca, Magdalena, Rancheria, San Jorge, and Tuira rivers basins; *Eigenmannia limbata* specimen was collected in the field in Suriname river and a *Sternopygus macrurus* tissue was from Parana (Argentina) belonging to the STRI collection ; for sampling see Appendix S1 (Supporting information). Total DNA was obtained from 20 mg of muscle with the BioSprint 96 from QIAGEN^®^ at The Smithsonian Tropical Research Institute (STRI). Following extraction, we quantified DNA extracts using a Qubit fluorometer (Life Technologies, Inc., Waltham, MA, USA) and samples were sent to MYcroarray, Inc (Ann Arbor, MI, USA) for a targeted sequencing approach. Probes were targeted (>200 base pairs) using target capture and Illumina sequencing (HiSeq 2500 PE100, Chicago, IL, USA). The capture target design is described in detail by Arcila et al. (2017). Although probes were not designed to target mtDNA genes, we tested whether the raw Illumina data contained mtDNA genomes.

We mapped reads from each sample to its most relative available mitochondrial genome of reference (*Eigenmannia sp*. (Genbank AB054131) for *Eigenmannia* individuals and *Sternopygus aequilabiatus* (unpublished) for *Sternopygus* individuals) using SAMTOOLS (v1.3.1; Li et al. 2009). Samples were mapped to reference, trimmed and the consensus sequences were obtained using GENEIOUS (v8.1.9; Kearse et al. 2012). A consensus sequence per each species was extracted from the multiple alignment and posteriorly were cleaned and visually inspected to detect stop codons. The mtDNA genome was annotated using MitoAnnotator (Iwasaki at al. 2013).

The mitochondrial genomes of *Eigenmannia humboldtii* (Genbank MH263668), *Eigenmannia limbata* (Genbank MH263669), and *Sternopygus macrurus* (MH263671) are complete, with 13 protein-coding genes, 1 D-loop, 2 ribosomal RNAs, and 22 transfer RNAs for a total length of 13,394, 10,921, and 13,013 bp, respectively. For *Sternopygus aequilabiatus* (MH263670) we couldn’t recover D-loop, this genome has 13 protein-coding genes, 2 ribosomal RNAs, and 22 transfer RNAs for a total length of 14,270 bp. Base compositions are typical for vertebrate mitochondrial genomes with 27.4% A, 29.1% C, 18.0% G, and 25.4% T for *E. humboldtii*, 27.5% A, 28.3% C, 18.7% G, and 25.4% T for *E. limbata*, 29.7% A, 27.8% C, 15.6% G, and 26.9% T, for *S. aequilabiatus* and 29.7% A, 27.4% C, 16.0% G, and 26.8% T for *S. macrurus*.

We reconstructed a phylogenetic tree based on the complete mitochondrial genome of 57 individuals belonging to the Sternopygidae family. The Bayesian tree was performed using MrBayes (v3.2.6; Huelsenbeck and Ronquist 2001; Ronquist and Huelsenbeck 2003) for 8 MCMC runs, 10 million of generations and sampled every 1000. Cis-andean species *E. limbata* and *S. macrurus* are both the sister group of the trans-Andean species; a remarkable find, is that individuals belonging to the great Magdalena basin falls in the same cluster (Figure 1).

**Figure 1.**
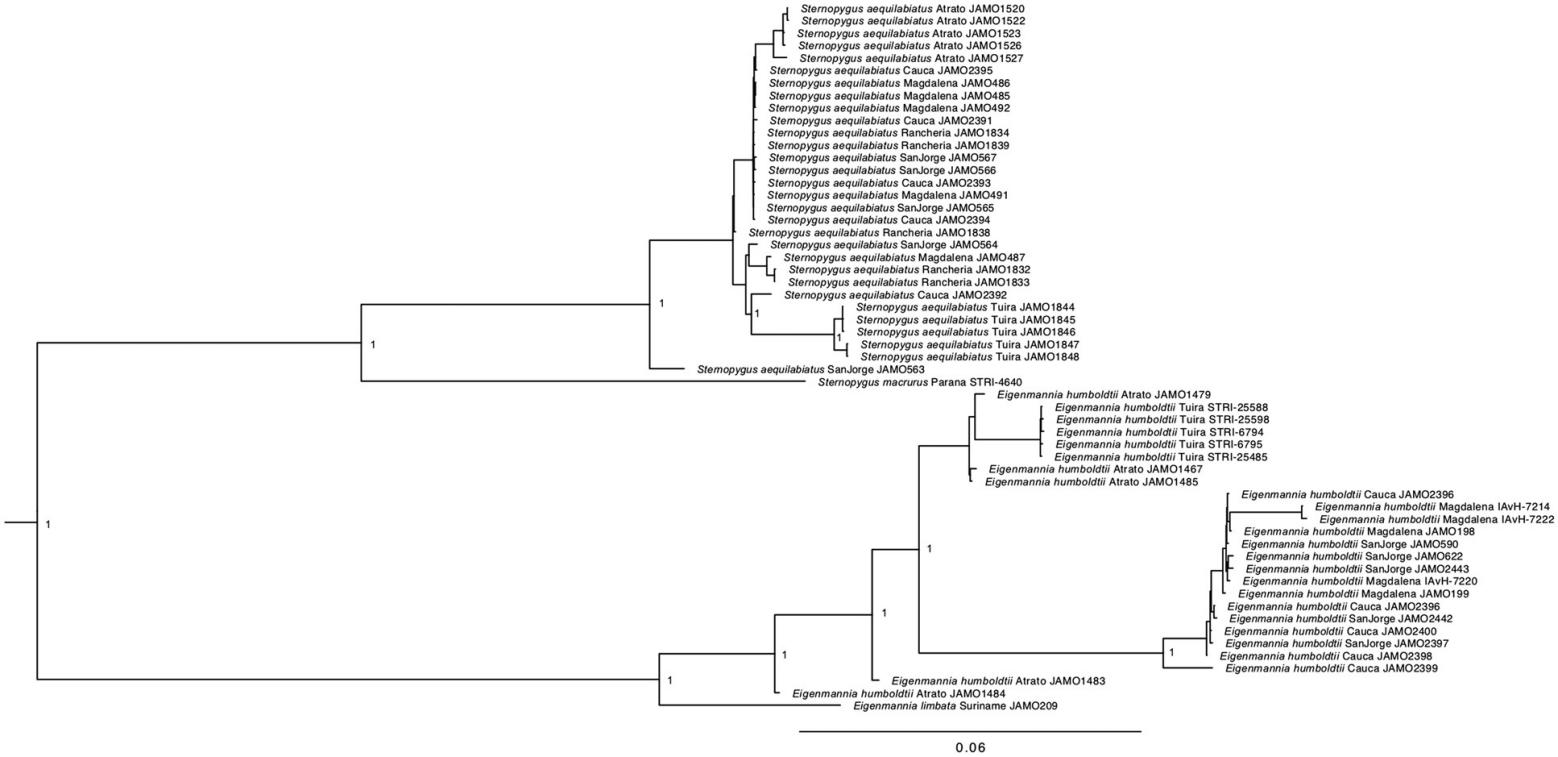
Molecular phylogeny of four South American electric knifefishes based on complete mitogenome. Support values at each node are Bayesian posterior probabilities. Branch label include information about sampled basin and tissue availability (JAMO: Pontificia Universidad Javeriana; IAvH: Instituto de Investigaciones Alexander von Humboldt; STRI: Smithsonian Tropical Research Institute).

## Supplementary Material

AppendixS1.xlsx
